# Real-Time Monitoring of Psychotherapeutic Processes: Concept and Compliance

**DOI:** 10.3389/fpsyg.2016.00604

**Published:** 2016-05-03

**Authors:** Günter Schiepek, Wolfgang Aichhorn, Martin Gruber, Guido Strunk, Egon Bachler, Benjamin Aas

**Affiliations:** ^1^Institute of Synergetics and Psychotherapy Research, Paracelsus Medical UniversitySalzburg, Austria; ^2^Department of Psychosomatics and Inpatient Psychotherapy, Christian Doppler University Hospital, Paracelsus Medical UniversitySalzburg, Austria; ^3^Faculty of Psychology and Educational Sciences, Ludwig Maximilians UniversityMunich, Germany; ^4^Institute of Psychoanalysis and Family TherapySalzburg, Austria; ^5^Complexity ResearchVienna, Austria

**Keywords:** real-time monitoring, momentary ecological assessment, nonlinear dynamics, compliance, process-outcome research

## Abstract

**Objective:** The feasibility of a high-frequency real-time monitoring approach to psychotherapy is outlined and tested for patients' compliance to evaluate its integration to everyday practice. Criteria concern the ecological momentary assessment, the assessment of therapy-related cognitions and emotions, equidistant time sampling, real-time nonlinear time series analysis, continuous participative process control by client and therapist, and the application of idiographic (person-specific) surveys.

**Methods:** The process-outcome monitoring is technically realized by an internet-based device for data collection and data analysis, the Synergetic Navigation System. Its feasibility is documented by a compliance study on 151 clients treated in an inpatient and a day-treatment clinic.

**Results:** We found high compliance rates (mean: 78.3%, median: 89.4%) amongst the respondents, independent of the severity of symptoms or the degree of impairment. Compared to other diagnoses, the compliance rate was lower in the group diagnosed with personality disorders.

**Conclusion:** The results support the feasibility of high-frequency monitoring in routine psychotherapy settings. Daily collection of psychological surveys allows for the assessment of highly resolved, equidistant time series data which gives insight into the nonlinear qualities of therapeutic change processes (e.g., pattern transitions, critical instabilities).

## Introduction

### Concept and criteria of high-frequency real-time monitoring

Outcome monitoring and feedback on therapeutic progress has become popular and has been adopted by many mental health providers all over the world (e.g., Howard et al., 1996; Evans et al., 2002; Kraus et al., 2005; Miller et al., 2005; Trauer, 2010; Schiepek and Aichhorn, 2013). Lambert (2007) or Newnham and Page (2010) describe it as an important feature of good clinical practice and ask for an integration of monitoring procedures into routines of mental health care (Lambert, 2010). This article supports the demand for a feedback-informed practice but also illustrates that its potentials are not yet exhausted. Some criteria are listed which can be combined to powerful synergies establishing a feedback-driven therapy approach. The feasibility of fulfilling these criteria will be demonstrated by reporting on compliance data from the application of an internet-based feedback technology (Synergetic Navigation System) to inpatient and day treatment settings.

### In session vs. ecological momentary assessment

Integrating feedback systems into psychotherapy remains an exception in today's clinical practice. The majority of those who do use feedback routines ask clients for outcome ratings during the actual therapy sessions (e.g., Lambert et al., 2002a; de Jong et al., 2014). Often, the administration of feedback questionnaires in inpatient or day treatment settings occurs infrequently and on a non-regular basis before or after a therapy session (Newnham et al., 2010a,b). Therapy feedback then loses the advantages of ecological momentary assessment, because experiences of every-day life aren't reported in close timely proximity to their actual occurrence. In contrast, daily assessment can reduce memory biases, distortions by state-dependent memory effects in distal settings, and the urge for implicit averaging over many events or days, resulting in enhanced ecological validity of the data (Fahrenberg et al., 2007; Ebner-Priemer and Trull, 2009; Wenze and Miller, 2010). For data collection in everyday settings, modern web-based devices such as smartphones, tablets, or laptops yield easy access to questionnaires whenever and wherever needed.

### Outcome vs. common factors monitoring

Feedback procedures focus almost exclusively on outcome measures. One widely used survey is the Outcome Questionnaire (OQ-45) developed by Lambert and colleagues (Lambert et al., 2004), which combines subscales of “symptom distress,” “interpersonal relations,” and “social role.” Other examples of feedback procedures using outcome measures are the WHO Wellbeing Index (WHO-5; Bech et al., 1996), the Mental Health subscales of the Medical Outcomes Questionnaire (SF-36; Ware et al., 1993), the Health of the Nation Outcome Scales (HoNOS; Wing et al., 1998), the Depression Anxiety Stress Scales (short version: DASS 21; Lovibond and Lovibond, 1995), and many more (see Evans et al., 2002; Howard et al., 1996; Trauer, 2010; Newnham et al., 2010a,b). Focusing entirely on—albeit important—outcome excludes process-mediating aspects and general therapeutic ingredients. In order to grasp these aspects of therapy, the monitoring should also cover client factors (resources, motivation to change, engagement), working alliance, emotions, self-relatedness, expectancies, self-esteem, self-efficacy, or ward atmosphere (Duncan et al., 2010; Norcross and Lambert, 2011). Thus, besides outcome, therapy feedback should also cover process-mediating factors and be sensitive to important features of change processes like early rapid responses, sudden gains or losses (Stiles et al., 2003; Lutz et al., 2013), or rupture-repair sequences in the working alliance (Stiles et al., 2004; Gumz et al., 2012a). Combining the common factors approach with therapy monitoring could result in a real-time assessment of common factor dynamics—with great importance to further research on this topic.

### Irregular vs. frequent and equidistant time sampling

**Figure 1:**
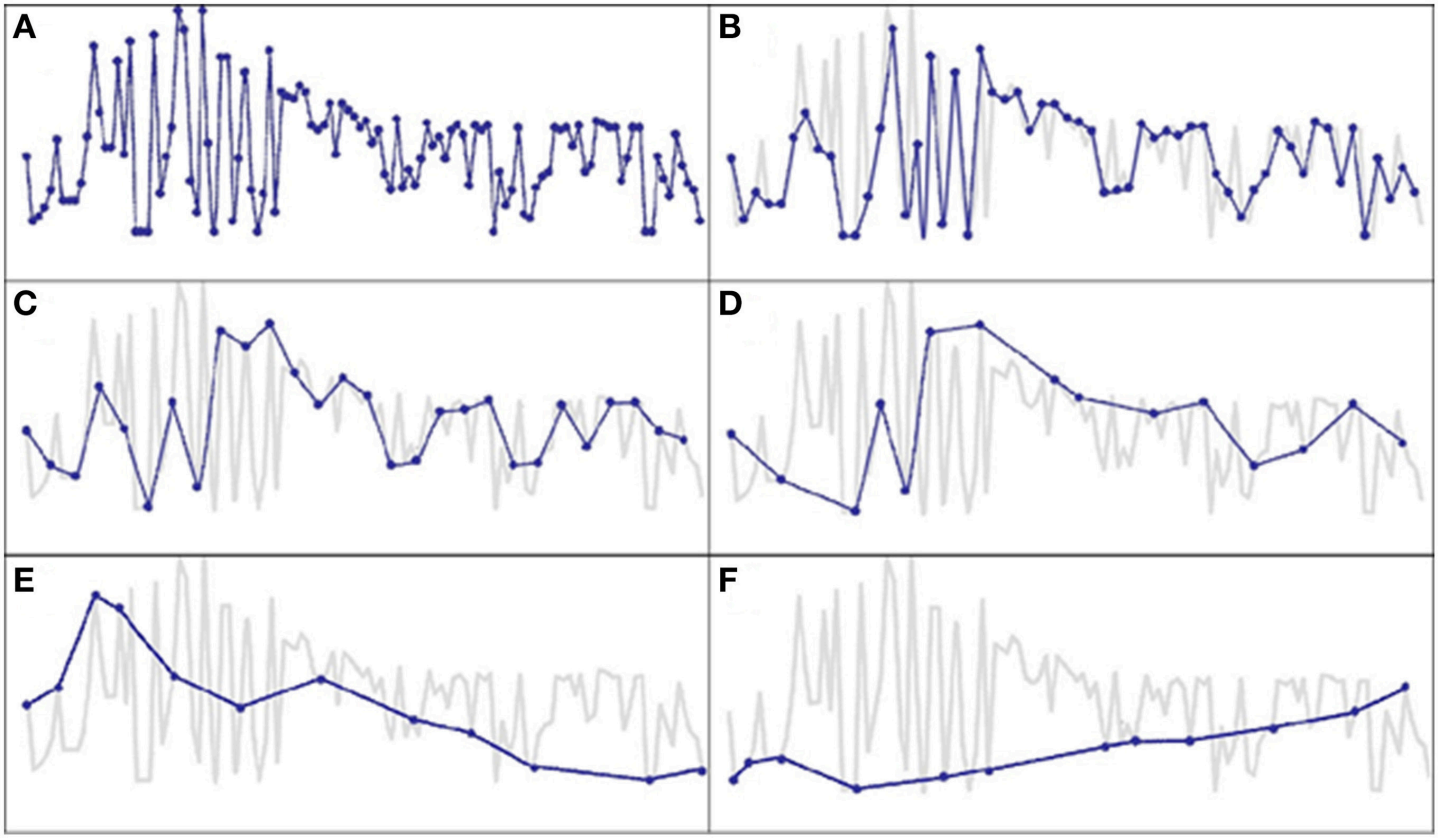
**Distortion of the dynamics of a time series by omitting measurement points**. Depicted is a self-esteem time series of a single client (with borderline personality disorder diagnosis). **(A)** Shows the original time series with daily responses (opaque in **B–F**). In **(B)** only every second day is omitted as missing day. Fluctuations of the first weeks of the time series vanish, if ratings are only made on every fourth day **(C)** or weekly with some variation **(D)**. A major loss of information and possible source of therapeutic misjudgment occurs with the common practice of occasional weekly and fortnightly measurement intervals **(E,F)**.

Frequent and regular assessment of psychological states and processes throughout therapy is not common in everyday practice. Often it is the sequence of therapy sessions that defines when patients give survey-based feedback. de Jong et al. (2014) report on a feedback study in outpatient settings with about 50% OQ administrations out of 32.3 (SD: 41.4) therapy sessions. de Beurs et al. (2011) administered the Brief Symptom Inventory four times during a sequence of more than 50 sessions. Such sampling rates represent outcome states at a certain time, but do not allow for the identification of dynamic patterns and pattern transitions. Figure 1 illustrates how the dynamics of a time series (daily ratings of self-esteem from a patient with Borderline Personality Disorder) is distorted and the information on the dynamic pattern is lost if measurement points are successively omitted. The rapid cycling of self-esteem (as well as emotions like grief, anger, or joy, not shown in this figure) characterizing the first weeks of a treatment, vanishes if ratings are only made on every fourth day (Figure 1C), weekly (Figure 1D), or at mixed weekly and fortnightly intervals, the most common periodicity of therapy sessions (Figures 1E,F). Corresponding to the loss of information, the dynamics of the presented time series appear more and more linear with the shape of the curve depending on the chosen measurement points.

In order to get deeper insight into human change processes, it is important to perform frequent, continuous, and equidistant measurements (regular time sampling). Only regular and frequent assessments through (process-) questionnaires allow for meaningful application of time series analysis methods in the frequency domain (e.g., Fast Fourier Transformations, Time-Frequency Distributions, Cohen, 1989) and particularly in the domain of nonlinear dynamics (Kantz and Schreiber, 1997; Heath, 2000; Haken and Schiepek, 2010).

The following thought experiment illustrates how crucial the relation is between the momentum of a phenomenon of interest (Eigendynamics) and the frequency of the measurement (sampling rate): Imagine a completely dark room, with a disk at the center, spinning continuously at one speed. The disk has a dot painted on it and the only source of light to determine the dot's position is a stroboscopic light. Depending on how frequently the light flickers, the dot's position, and thus the rotational speed of the disk, appears to be very different. If the light matches the disk's rotation frequency exactly, the disk appears to be static; however, depending on the frequency of the flickering light, the disk and dot can just as well appear to be moving quickly, slowly, forwards, backwards, or completely erratically. So even for a regularly and continuously spinning dot, a non-standardized and infrequent detection method can only yield invalid results. Going one step further, imagine we tried to estimate the behavior of a dot painted on a chaotically moving double pendulum. In that case, not only the measurement frequency is irregular, but also the dynamic of the phenomenon under investigation is unknown/chaotically. The only way to give a rough estimate of the true behavior of the double pendulum in that imagined dark room would be to install a very rapidly flickering stroboscope and it needs to do so very regularly. In analogy to this phenomenon, if a psychological process is in any form nonlinear or susceptible to sudden changes, the only way to detect—and feedback—theses changes is via frequent and regular measurement. Only then intra-personal identification of processes can be achieved and put to direct use in psychotherapy. In consequence, there should be just as much emphasis placed on standardizing the frequency of measurement as there is currently on standardizing the instruments used for measurement (e.g., questionnaires).

When aiming at (a) a complete recording of therapies (not only as an irregular event sampling), (b) frequent and (c) continuous measurements, and (d) considering practicalities of data collection, daily measurements appear to be a good and achievable way. The present paper therefore reports on results from daily administration of a process questionnaire.

### Linear vs. nonlinear dynamics

Most therapy feedback applications utilize linear models of psychological change. However, there are accumulating findings supporting nonlinearity and chaoticity of psychotherapy and change dynamics (e.g., Kowalik et al., 1997; Schiepek et al., 1997, 2014a,b; Tschacher et al., 1998; Hayes et al., 2007a,b; Granic et al., 2007; Haken and Schiepek, 2010; Gumz et al., 2012b; Heinzel et al., 2014). Chaos implies different degrees of irregularity and complexity of the dynamics, including its sensitive dependency on initial conditions, on minimal input onto the system, or on micro-fluctuations (Schuster, 1989; Strunk and Schiepek, 1996). This so called “butterfly effect” restricts the predictability of systems' behavior dramatically.

Another well-known feature of human change processes is phase-transition-like behavior as modeled by theories of self-organization (especially Synergetics, Haken, 2004; Haken and Schiepek, 2010; Schiepek et al., 2014a,b, in press). Sudden changes (gains or losses) during psychotherapies may directly correspond to such phase transitions. It should be mentioned that pattern transitions can be found at the mean data level of a time series, but also in their variability, rhythms, frequency distribution, complexity, or other dynamic features (see Figure 1A). Synergetics, e.g., predicts the occurrence of critical fluctuations and the increase of data-variability just before transitions from one pattern to another take place (Kelso, 1995; Haken, 2004; Haken and Schiepek, 2010; Schiepek et al., 2014a,b, in press).

**Figure 2:**
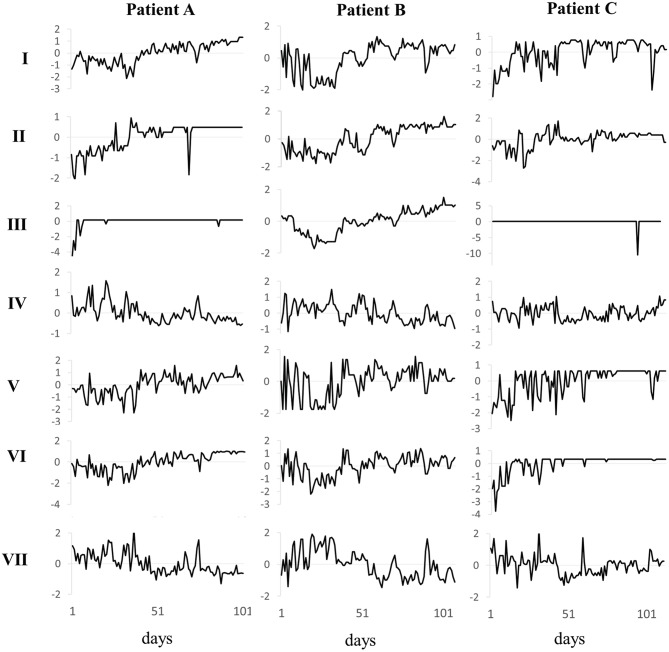
**Time series of seven TPQ factors of three patients with non-linear tracks**. Depicted are the seven z-transformed factors of the daily administered Therapy Process Questionnaire of three patients. Only through high resolution administration of questionnaires it becomes evident, that patient A (F33.2, recurrent depressive disorder, current episode severe without psychotic symptoms), patient B (F42, obsessive-compulsive disorder), and patient C (F60.3, emotionally instable personality disorder) not only differ in terms of pre- and post-values of the factors, but also the shape, range, fluctuation, and time points of order transitions differ.

Both, critical fluctuations at instability points of the system dynamics and the deterministic chaos of the process—confounded with stochasticity in real-world systems—result in high complexity and inter-individual diversity of dynamics. From the point of view of complex dynamic systems, standard tracks (expected change trajectories) are more likely to be an artifact of infrequent/irregular data collection and widely used linear assumptions, than of the actual linearity of the researched phenomena. In other words, daily measurements using an appropriate survey (e.g., the Therapy Process Questionnaire, Schiepek et al., 2012), yield time series of psychotherapies that allow to capture and identify diversity and complexity of cases, as well as critical instabilities and nonstationarities (pattern transitions), as Figure 2 exemplifies for two patients.

Unpredictability and complexity of change processes thus make close monitoring important. If all therapies of a certain type (e.g., the same diagnosis) followed the same expected trajectory and the dynamics could be controlled by the input (i.e., the interventions), it would be a safe bet to recommend invariant manuals. However, the more the courses of psychopathology and recovery deviate from linear input-output mechanisms and from planned routes, the more we need immediate support by feedback systems that provide information at short time delays to the ongoing process. Time series analysis tools offer just that, by reporting on nonlinear features of processes such as varying complexity, critical fluctuations, time-dependent synchronization of signals, and other precursors of pattern transitions (Scheffer et al., 2009; Molenaar, 2010).

### Focus on cases at risk of deterioration vs. participative process control by applying decision rules to all cases

There is increasing evidence that feedback not only supports therapy in cases of threatening deterioration (Lambert et al., 2002b) but also in prosperous therapies (Lambert et al., 2005; Anker et al., 2009; de Jong et al., 2014). It appears to be especially beneficial if both, client(s) and therapist exploit feedback (Hawkins et al., 2004; de Jong et al., 2014). In consequence, feedback tools should become part of everyday routine practice in different psychotherapeutic settings (outpatient, inpatient, day treatment centers, home-based treatment, counseling) and the information produced should be shared by clients and therapists. Feedback-based therapy sessions not only focus on potentially poor outcome or the application of clinical support tools to prevent deterioration (Lambert et al., 2005); also a diversity of dynamic features shift into focus, and, as predicted by the theory of self-organization, critical instabilities, and crises are utilized as common and necessary transients on the way to therapy effects. Therapists should be able to read these markers of self-organizing processes and encourage the client to communicate his/her experiences corresponding to the feedback results. In consequence, clients will be accompanied toward further therapeutic steps and strengthened for (micro-)decisions on the way to therapeutic success. Herein the therapist continuously realizes a threefold reference: (i) to the information given by the client, (ii) to the theory (e.g., the theory of self-organization), and (iii) to the process data and analysis results (Schiepek et al., 2015).

To summarize, the need for ecological momentary assessment that takes into account common factors of psychotherapy asks for frequent and equidistant time sampling. Nonlinear dynamics can be detected and via feedback integrated into the cooperative work of client and therapist. Besides underlining the conceptual framework, we specifically aim at reporting on the possibility and difficulties of administering questionnaires on a daily basis. It is of particular interest, how many days patients miss to fill in throughout a therapy cycle of 50 and 90 days. In addition, the representative sample of the year 2013 at the University Hospital Salzburg allows to report on the distribution of missing days and their relationship with specific diagnoses, severity of symptoms and differences between in- and outpatient treatment. The results will allow to draw conclusions about the possibility of conducting nonlinear time series analyses to psychological data in future research and mathematical modeling (Bornas et al., 2014; Schiepek et al., in press).

## Materials and methods

### The synergetic navigation system (SNS)—an internet-based feedback technology

Data collection and real-time monitoring was realized by the Synergetic Navigation System (SNS). SNS is a web-based generic system that allows for the implementation of various questionnaires at any chosen interval. The response options to the items combine Likert-type scales and visual analog scales. Data can be entered using web-compatible devices including PCs, notebooks, tablets, or smartphones, which permits maximal spatial and temporal flexibility for entering data. Data privacy protection and data security are guaranteed by https-pages, anonymized usernames, passwords, and security technology at the level of online banking transactions. Alongside an administrative mode and client documentation, a range of outcome and process questionnaires can be selected. The raw data results can be visualized by time series graphs. These time series can be submitted to several analyses, e.g., Dynamic Complexity (Schiepek and Strunk, 2010), Recurrence Plots (Eckmann et al., 1987; Webber and Zbilut, 1994), Permutation Entropy (Bandt and Pompe, 2002), or the visualization of synchronization patterns by item-to-item intercorrelations calculated in a running window. All types of analysis are open to be used by therapists and can be integrated into feedback and used for individualizing therapeutic decisions.

### Questionnaires

As daily process monitoring, patients filled in the Therapy Process Questionnaire (TPQ, Haken and Schiepek, 2010; Schiepek et al., 2012; see Appendix in Supplementary Material for an English translation of the TPQ). The 42 items of the inpatient version are given on seven-point Likert scales or visual analog scales, and the continuous ratings are transformed into time series of the clinical course. All items request an answer, so the clients are not able to skip questions. A factor analysis resulted in a 7 factor resolution: I Therapy progress, confidence in treatment effects, self-efficacy; II Ward atmosphere, social relationship to fellow patients; III Working alliance and trust in therapists; IV Dysphoric emotions; V Opening of perspectives, insight, personal innovations; VI Intensity of therapeutic work, motivation to change; VII Impairment by symptoms and problems (see Figure 2 and Appendix in Supplementary Materials). The application of the feedback system started in 2007 and continues. A second explorative factor analysis combined with a confirmatory factor analysis of the items revealed a 5 factor structure (Schiepek et al., 2012).

For an assessment of pre-post differences and follow-ups, the ICD-10 Symptom Rating is used (ICD-10-SR; Tritt et al., 2007). Clients reported weekly on the respective scales of the Depression Anxiety Stress Scales (short form: DASS 21; Lovibond and Lovibond, 1995; Antony et al., 1998; Newnham et al., 2010b). In addition, several questionnaires are restricted to specific diagnoses (e.g., Borderline Symptom List [BSL]; Bohus et al., 2009; Yale-Brown Obsessive Compulsive Scale [Y-BOCS]; Goodman et al., 1989) or are used in specific projects only (e.g., the German version of the Outcome Questionnaire [OQ]; Lambert et al., 2002b, 2004).

### Participants

This feasibility study was realized at the Department of Inpatient Psychotherapy and at the Department of Psychosomatics/Day-Treatment Center, Christian Doppler University Clinic, Salzburg, Austria. The sample includes the cohort of 159 clients which were released after treatment in 2013 [7 clients were not included in the SNS routine, 1 was excluded for inconsistencies in the data set; see Table 1 for a characterization of the sample (*N* = 151)]. The Therapy Process Questionnaire is filled in daily, normally in the evening hours. Its welcoming text specifically asks to report on all aspects covered in the TPQ as experienced at the current day. Clients were free to access the questionnaires via internet through private devices or make use of the clinic's infrastructure. The introduction to the TPQ items and the use of SNS is made by the responsible psychotherapist at the very beginning of the hospital stay. All clients sign an informed consent, introducing real-time monitoring as a routine part of the clinics practice as well as the willingness for allowing the data to be used for empirical purposes. Application of the SNS to patients and the usage of the retrieved data has been approved by the ethical committee of the Paracelsus Medical University.

**Table 1:** **Descriptives, comorbidity and baseline DASS-21 and ISR-Total scores**.

	***n***	**Mean**	**SD**
Discharged in 2013	159		
Included in data analysis	151^*^		
Inpatient psychotherapy	115		
Day-treatment center	37		
Female/Male (%)	107 (70.4%)/45 (29.6%)		
Age		36.2	11.6
Patients with/without Comorbidity (N, %)	96 (63.8%)/55 (36.2%)		
Comorbidity (mean additional diagnoses)		1.05	1.00
DASS-21 Depression		21.52	11.34
DASS-21 Anxiety		14.69	8.61
DASS-21 Stress		21.19	8.95
ISR Total Score		1.62	0.56

* *7 clients were not included in SNS, while the data of 1 client was not useable due to technical problems*.

### Compliance, completeness, and missing data

Compliance for filling out the process questionnaire (TPQ) once per day was defined by the percentage of days a client answered the questionnaire related to the days of his/her hospital stay. Delayed starting, early termination, or missing data within the monitoring period all reduce the compliance rate. The maximum number of treatment days is defined by the regular treatment period of 8 weeks in the day-treatment unit of the Department of Psychosomatics and 12 weeks in the Department of Inpatient Psychotherapy (hereof some patients sleep at home and can therefore be understood as day-treatment patients of that department). All—but one—patients, that started to fill the TPQ at a respective day, also finished all items of that day's questionnaire. There are no further questionnaires with single missing items. Therefore, missing data can be understood as missed days, expressed as percentage of missed days in relation to all days of SNS-activation. Completeness relates to the percentage of days patients actually fulfilled the planned treatment stay. 100% completeness corresponds to a treatment cycle of 50 days in the Department of Psychosomatics and of 90 days in the Department of Inpatient Psychotherapy. If the client stayed longer—a procedure occasionally chosen when clinically indicated—he/she is defined as a 100% completer, too. Less than 100% completeness may have various reasons, e.g., aversion to the treatment concept, acute suicidality, discharge for disciplinary reasons, exacerbation of psychotic symptoms, or drop out by a client's decision to finish the treatment.

To answer the research question of a possible relationship between symptom severity and compliance rate, the correlation between the subscales of the DASS and the ISR total score and the compliance rate were calculated. The dependency of the compliance rate on the superordinate ICD-10 diagnosis categories F30, F40, and F60 was tested by a univariate ANOVA with diagnosis categories as the independent and the compliance rate as the dependent variable.

## Results

### Compliance and completeness

**Figure 3:**
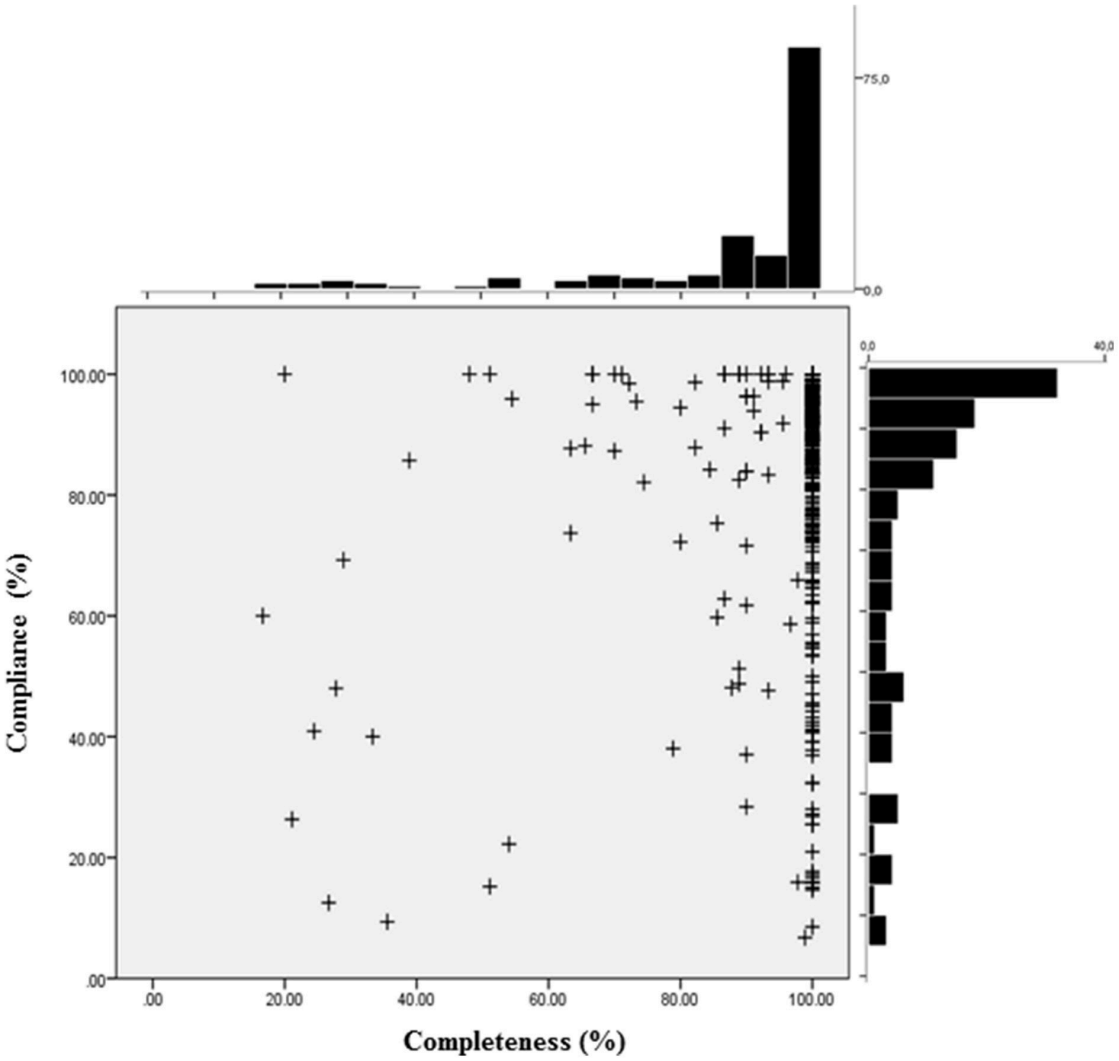
**Distribution of response compliance and treatment completeness**. The scatterplot depicts for each patient (+) the individual compliance and completeness score. Compliance denotes the percentage of days a client answered the questionnaire per number of days of his/her actual hospital stay. Completeness is defined as the percentage of days a client fulfilled the planned treatment stay (50 or 90 days, respectively).

The mean compliance of the sample was 78.3% (SD 26.0; median 89.4%). 66.4% of all clients realized a compliance rate of 80% or more, 49.3% realized a compliance rate of 90% or more, and 25 clients (16.4%) had a compliance rate of 100%. Figure 3 illustrates the distribution of the clients in a compliance-completeness scatter plot. 37.5% of all patients made use of at least 90% of the available treatment period (completers) and had a compliance of at least 90% to the daily real-time monitoring. Of all clients with a treatment completeness of ≥ 90% (*N* = 107), only 21 (13.8%) had a compliance rate of less than 60%, and only 32 (21.1%) filled in the daily questionnaire less than 80% of the treatment days. Treatment completers thus realized a high rate of monitoring compliance. Clients with inpatient treatment (*n* = 58) had significantly less compliance as compared to patients in day treatment (*n* = 87), with *t*_(143)_ = −1.98, *p* = 0.049, [95% *CI*: −16.9 – −0.02].

### Distribution of missing data

**Figure 4:**
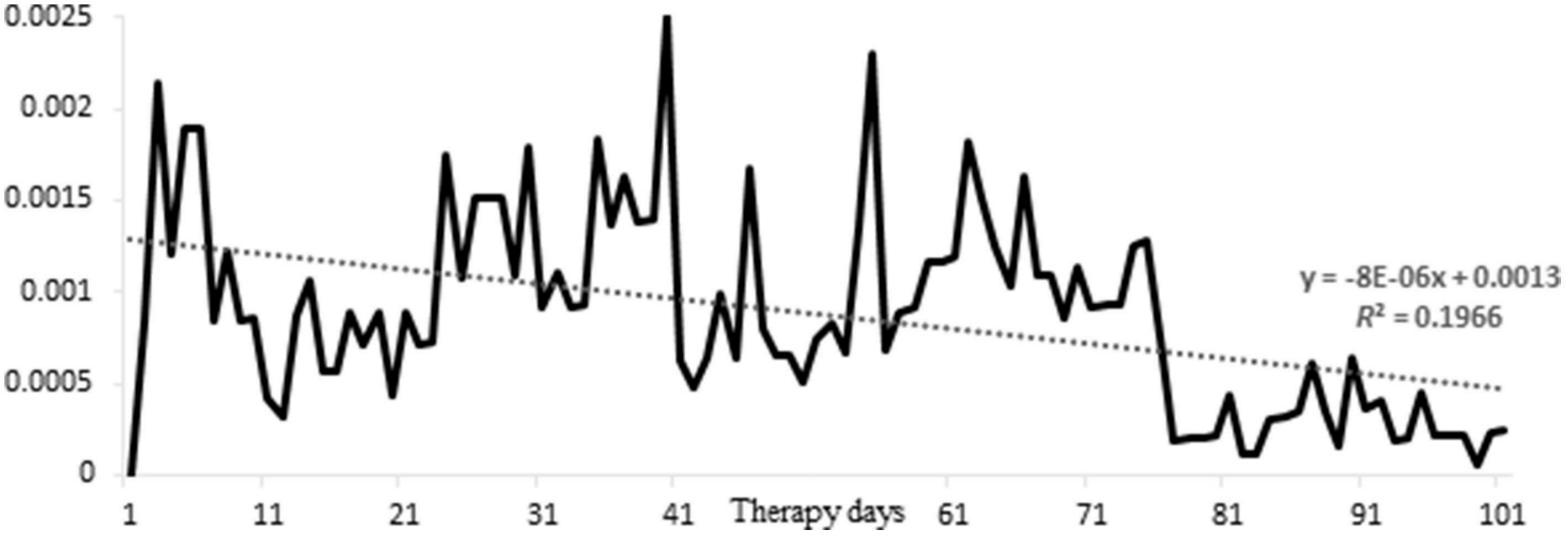
**Distribution of missed days**. Depicted is per day the squared number of missed days, divided by the product of total possible number of clients missing a day X the total number of missed days in the sample; one can interpret the figure as the distribution of missed days, corrected for the fact that the sample thins out toward increasing number of therapy days.

The mean time series length of the 115 clients of the Department of Inpatient Psychotherapy was 73.4 measurement points (= days) (SD 31.5), the mean time series length of the 37 clients of the psychosomatic day-treatment center was 47.9 (SD 10.0). The complete sample had on average 10.1% missing values within the time series (SD 18.0; median 1.3%) (inpatient psychotherapy: 10.3%, SD 19.3, median 0.0%; day-treatment center: 9.4%, SD 13.2, median 3.9%). 81.6% of all clients had less than 20% missing data, 73.7% of all clients had less than 10% missing data, and 74 clients (48.7%) had no missing data (0%). Figure 4 shows the distribution of missing days in relation to the number of patients in treatment, corrected for the total number of missing days in the sample. The decreasing trend of this parameter exemplifies that patients that started SNS become less likely to miss a day, the longer they stay in treatment. With other words, the data suggests that patients do not get tired to fill in a questionnaire on daily basis. Consequently, there is a positive relationship between length of hospital stay and monitoring compliance, expressed by a correlation of 0.231 (*p* < 0.004).

### Compliance and its relationship to symptom severity

Symptom or problem severity was defined by the DASS subscales (depression, anxiety, and stress) and the ISR total score at admission to psychotherapy. The Pearson correlation coefficients of the compliance rate with these scales are as follows: DASS depression scale: *r* = −0.081 (*p* = 0.325), DASS anxiety scale: *r* = −0.035 (*p* = 0.668), DASS stress scale: *r* = −0.069 (*p* = 0.403), ISR total score: *r* = −0.073 (*p* = 0.380). This corresponds to a real null correlation between symptom/problem intensity or burden of disease with the monitoring compliance.

### Compliance and its relationship to type of diagnosis

For testing the differences between the compliance rates of the frequent diagnosis categories of this sample [F30: mood (affective) disorders; F40: neurotic, stress-related, and somatoform disorders; F60: disorders of adult personality and behavior; see Tables 1, 2] a univariate ANOVA was calculated (*F* = 5.37, *df* = 2, *p* = 0.006). Multiple comparisons (Scheffé Test) indicate significant differences between the compliance rates of F30 (83.2%) and F60 (66.1%) (*p* = 0.010), F40 (81.1%) to F60 (66.1%) (*p* = 0.027), but not between F30 and F40 (*p* = 0.920). Evidently, the compliance rate of the clients diagnosed with personality disorders (F60) was significantly lower than the compliance rate of the other categories (F30, F40). A comparison of the diagnosis categories with % missing data (Table 2; univariate ANOVA) produced similar but less pronounced results (*F* = 3.26, *df* = 2, *p* = 0.041). Multiple comparisons (Scheffé Test) indicate that the difference between the percentage of missing data of F30 (6.9%) and F60 (16.3%) scarcely misses the 5%-significance level (*p* = 0.051), differences between F40 (8.6%) and F60 (16.3%) (*p* = 0.136), or between F30 to F40 (*p* = 0.882) are far from being significant.

**Table 2:** **Diagnoses of the sample and their prevalence, percentage in sample, percentage compliance and percentage missing data**.

***ICD -10 diagnostic category***	***n***	**Percentage of sample**	**Percentage compliance^c^**	**Percentage missing days^d^**
		**%**	**Mean (SD)**	**Mean (SD)**
F10^a^ Mental and behavioral disorders due to psychoactive substance use	1	0.7	100	−
F20^a^ Schizophrenia, schizotypal, and delusional disorders	4	2.6	75.1 (23.9)	2.4 (4.8)
F30 Mood (affective) disorders	53	34.9	83.2 (23.9)	6.9 (14.6)
F40 Neurotic, stress-related, and somatoform disorders	53	34.9	81.1 (24.1)	8.6 (13.4)
F50^a^ Behavioral syndromes associated with physiological disturbances and physical factors	4	2.6	77.4 (25.9)	22.2 (24.6)
F60^b^ Disorders of adult personality and behavior	36	23.7	66.1^*^ (29.7)	16.3 (25.7)
Total^e^	151	100	78.3 (26.0)	9.9 (17.9)

a *Not included into ANOVA*.

b *of which n = 31 are diagnosed with F60.3 Emotionally instable personality disorder*.

c *Percentage compliance is relative to the intended treatment length of 50 or 90 days*.

d *Percentage missing data is number of filled in days relative to the absolute treatment length*.

e *For 1 patient, diagnoses were not recorded*.

* *significant at p = 0.041*.

## Discussion

The results illustrate the feasibility of a highly resolved real-time monitoring procedure on psychotherapy processes in an inpatient and a day-treatment setting. A large proportion of the clients revealed high compliance rates and low rates of missing data, resulting in time series of high frequency, low missing rates, and equidistant measurements. Application of the questionnaires was accomplished using an internet-based device (SNS) with a process questionnaire of 42 items (Therapy Process Questionnaire, Haken and Schiepek, 2010) during the complete period of psychotherapy of at least three respectively 2 months. Problem or symptom severity had no impact on the compliance rate, which implicates that more impaired patients can also make use of the monitoring procedure. Clients with personality disorder diagnosis [F60, most of them (31 out of 36) emotionally instable personality disorder, F60.3] revealed lower rates of monitoring compliance and slightly higher rates of missing data (difference scarcely over the significance threshold) than others. The positive correlation between completeness (days of treatment) and compliance (*r* = + 0.231) indicates that there is no fatigue, resistance, or reactance effect with increasing duration of the monitoring (as a negative correlation would have suggested).

The study replicates the promising compliance rates found in other studies on ambulatory assessement (e.g., Voelkl and Mathieu, 1993; Axelson et al., 2003; Tennen et al., 2006; Knowles et al., 2007; Putnam and McSweeney, 2008; for a review see Wenze and Miller, 2010) and contradicts low compliance or acceptability rates reported especially for mood disordered clients (Barge-Schaapveld et al., 1999; Myin-Germeys et al., 2003). In a suicide prevention program high-freqeuncy monitoring was possible even with persons at high risk for suicide (Schiepek et al., 2011; Fartacek et al., 2016). In accordance with other studies, we found no reduced compliance at higher levels of symptomatology (Voelkl and Mathieu, 1993; Stetler et al., 2004; Peeters et al., 2006; Havermans et al., 2007). Further investigation is needed on the impact of repeated self-ratings on daily moods (Barge-Schaapveld et al., 1995). In comparison to the context of research studies on ambulatory assessment, real-time monitoring was used here in a therapeutic context for reasons of progress feedback and reflection on change dynamics. Using a monitoring questionnaire for therapy that reflects factors contributing to therapeutic progress activates personal resources, enhances motivation, supports re-moralization, and inspires confidence. A randomized controlled trial on the add-on effects of high-frequency monitoring and feedback (compared to treatment as usual without feedback) in chronic alcohol-dependent men showed effects in emotion perception and emotion regulation, but not in depression and other psychopathological symptoms (Patzig and Schiepek, 2015).

A limitation is the restriction of the data to inpatient and day-treatment settings. In consequence, an important future challenge will be the application to outpatient psychotherapy and to establish a feedback-driven dynamic systems approach in the routine practice of different settings and mental health services. Another challenge is the integration of the feedback-driven dynamic systems approach into the training of psychotherapists. An understanding of complex nonlinear systems will be of importance, since high-frequency monitoring and related tools for data analysis (like the SNS) make evidence for nonlinear processes and self-organization in every single case (Schiepek et al., 2015). A further component of training in psychotherapy monitoring is the practice of feedback-based interviewing and process reflection. The dynamics have to be reflected on regularly and implications for the next steps of the psychotherapeutic process have to be considered. Many years of practice and an actual interview study (unpublished data) gave evidence for the importance of the frequency and quality of feedback-referred interview sessions and its impact on the compliance and commitment to the monitoring.

The consequences for psychotherapy research are manifold: highly resolved real-time monitoring can define a standard for the investigation of dynamic phenomena like drop-outs and its precursors, sudden changes or sudden losses, crisis-repair sequences, phase-transition-like phenomena, dosis-to-effect shapes, or sustainable and long-term outcomes. Even the outcome may be evaluated not only by pre-post differences in outcome measures, but by changing dynamic patterns of cognitions and emotions [e.g., changes in the rapid cycling of mood or affective instability in Borderline Personality Disorders (compare Figure 1) or Bipolar Disorders, see Marwaha et al., 2014]. Here, the use of equidistant time-sampling is crucial. Studies on non-stationarities in neural networks during psychotherapy use the identification of critical instabilities by high-frequency monitoring to correlate neural and mental (cognitive/affective) pattern transitions (Schiepek et al., 2009, 2013). In the future, nonlinearity and nonstationarity of change processes can be assessed in ongoing psychotherapies, which should take more attention to sampling rates and the quality of monitoring based feedback to the clients.

One additional future application and improvement of feedback procedures are idiographic approaches to individual item selection and case formulation (Schiepek, 2003). Integrating personal topics, problems, and goals of a client to a questionnaire can improve the items' specificity and sensitiveness and thereby add to the validity of the information produced by the measurement instrument (Mumma, 2004; Barlow and Nock, 2009; Molenaar, 2013). Furthermore, clients will not only experience personal involvement; after editing an individual questionnaire together with a therapist, the professional relationship between therapist and client will also improve (unpublished data from an interview study, publication in preparation). This enhances the autocatalytic effects produced by continuous self-ratings and the compliance for filling out the questionnaires in short intervals. One approach to individualized monitoring is a systemic case formulation by idiographic systems modeling (Schiepek et al., 2015) which results in a graphic network of recursively interconnected variables constituting the most important cognitive, emotional, and social components of the client's problem system. This modeling work is done in close cooperation of client and therapist. The variables of the model are used to define the items of a personal questionnaire which can be designed by the questionnaire editor of the Synergetic Navigation System. Monitoring the results of a daily administered individualized questionnaire allows for the continuous assessment of a patient's idiosyncratic dynamics of therapy and for feedback to therapist and patient in time, thereby creating a powerful, feasible tool on basis of a feedback-driven dynamic systems approach.

## Author contributions

GSch and BA contributed to data gathering, text, study design, and data analysis. GSt contributed significant to data analysis and study design. EB, MG, and WA contributed significantly to data gathering and study design. All authors added corrections and proofread the paper.

## Funding

Paracelsus Medical University, Salzburg, Austria. Award number: R-14/02/058-AAS.

### Conflict of interest statement

The authors declare that the research was conducted in the absence of any commercial or financial relationships that could be construed as a potential conflict of interest.
