# Supplementary material for: Real-Time Monitoring of Psychotherapeutic Processes: Concept and Compliance
Source: Front Psychol. 2016 May 3;7:604. doi: 10.3389/fpsyg.2016.00604 (PMC4853656; doi:10.3389/fpsyg.2016.00604)
Supplement: Supplementary file 1 [file DataSheet1.docx]

**Appendix**

Therapy Process Questionnaire

| **Factor** | **Item-Nr. (*recode)** | **Items of the**  **Therapy Process Questionnaire (TPQ)** | **Scale and dimension extrema** |
| --- | --- | --- | --- |
| Therapeutic progress  /  Confidence  /  Self-Efficacy | 1 | *I am managing to solve my problems more and more.* | 0 – 1 – 2 – 3 – 4 – 5 – 6  not at all very much |
|  | 2 | *I now feel up to situations that I didn’t feel up to before.* | 0 – 1 – 2 – 3 – 4 – 5 – 6  not at all very much |
|  | 3 | *I now understand myself and my problems better.* | 0 – 1 – 2 – 3 – 4 – 5 – 6  not at all very much |
|  | 4 | *Today, I came closer to the solution to my problems.* | 0 – 1 – 2 – 3 – 4 – 5 – 6  not at all very much |
|  | 5 | *Today I was confident that I will solve my problems.* | 0 – 1 – 2 – 3 – 4 – 5 – 6  not at all very much |
|  | 6 | *Concerning my personal goals, I perceived myself as … today.* | 0 – 1 – 2 – 3 – 4 – 5 – 6  unsuccessful successful |
| Intensity of Treatment | 7 | *Today I worked on issues that really mean something to me.* | 0 – 1 – 2 – 3 – 4 – 5 – 6  not at all very much |
|  | 8 | *Today I was motivated to work on solutions to my problems.* | 0 – 1 – 2 – 3 – 4 – 5 – 6  not at all very much |
|  | 9 | *Today I dared to look into worrisome aspects of my life.* | 0 – 1 – 2 – 3 – 4 – 5 – 6  not at all very much |
|  | 10 | *After today’s experiences, I am determined to tackle my problems.* | 0 – 1 – 2 – 3 – 4 – 5 – 6  not at all very much |
|  | 11 | *Today I was interested/curious about the content and aspects of therapy.* | (visual analog slider)  not at all very much |
| New aspects  /  Innovation | 12 | *Today I became aware of how things are connected in a way, I hadn´t thought of before.* | 0 – 1 – 2 – 3 – 4 – 5 – 6  not at all very much |
|  | 13 | *New perspectives opened up for me today.* | 0 – 1 – 2 – 3 – 4 – 5 – 6  not at all very much |
|  | 14 | *Today I worked on things that were new and unusual for me.* | 0 – 1 – 2 – 3 – 4 – 5 – 6  not at all very much |
| Quality of relationship  /  Openness  /  Trust towards therapists | 15 | *I perceive the work with my therapist(s) as helpful.* | 0 – 1 – 2 – 3 – 4 – 5 – 6  not at all very much |
|  | 16 | *The therapist(s) ask helpful questions and give important suggestions.* | 0 – 1 – 2 – 3 – 4 – 5 – 6  not at all very much |
|  | 17 | *I feel comfortable in my relation with my therapist(s).* | 0 – 1 – 2 – 3 – 4 – 5 – 6  not at all very much |
|  | 18 | *I can be frank and honest towards the therapist(s).* | 0 – 1 – 2 – 3 – 4 – 5 – 6  not at all very much |
|  | 19* | *The therapists think differently about me than what they say.* | 0 – 1 – 2 – 3 – 4 – 5 – 6  not at all very much |
|  | 20* | *A different therapeutic approach would be better suited for me.* | 0 – 1 – 2 – 3 – 4 – 5 – 6  not at all very much |
| Climate and  Atmosphere at the clinic | 21 | *In the clinic I feel safe and supported.* | 0 – 1 – 2 – 3 – 4 – 5 – 6  not at all very much |
|  | 22 | *I can trust the other patients.* | 0 – 1 – 2 – 3 – 4 – 5 – 6  not at all very much |
|  | 23 | *I can be frank and honest towards the other patients.* | 0 – 1 – 2 – 3 – 4 – 5 – 6  not at all very much |
|  | 24 | *I feel comfortable with the other patients.* | 0 – 1 – 2 – 3 – 4 – 5 – 6  not at all very much |
|  | 25* | *In contact with the other patients, I feel tense.* | 0 – 1 – 2 – 3 – 4 – 5 – 6  not at all very much |
| Dysphoric affect  /  Inward orientation | 26 | *Today I felt sad.* | (visual analog slider)  not at all very much |
|  | 27 | *Today I felt anger.* | (visual analog slider)  not at all very much |
|  | 28 | *Today I felt guilt.* | (visual analog slider)  not at all very much |
|  | 29 | *Today I felt anxious.* | (visual analog slider)  not at all very much |
|  | 30* | *Today I felt self-confident.* | (visual analog slider)  not at all very much |
|  | 31* | *Today I felt joy.* | (visual analog slider)  not at all very much |
|  | 32 | *Today I felt shame.* | (visual analog slider)  not at all very much |
|  | 33 | *Today I felt hatred.* | (visual analog slider)  not at all very much |
|  | 34* | *Today I felt compassion.* | (visual analog slider)  not at all very much |
|  | 35 | *Today I dealt intensively with my feelings.* | 0 – 1 – 2 – 3 – 4 – 5 – 6  not at all very much |
|  | 36 | *Today I was quite insecure.* | 0 – 1 – 2 – 3 – 4 – 5 – 6  not at all very much |
| Intensity of Problems | 37 | *Today my problems were…* | (visual analog slider)  absent very strong |
|  | 38 | *Today my problems bothered me.* | 0 – 1 – 2 – 3 – 4 – 5 – 6  not at all very much |
|  | 39 | *Today my problems affected my daily life.* | 0 – 1 – 2 – 3 – 4 – 5 – 6  not at all very much |
|  | 40 | *Today I felt helpless and at the mercy of my problems.* | 0 – 1 – 2 – 3 – 4 – 5 – 6  not at all very much |
|  | 41 | *Today I avoided situations that are connected to my problems.* | 0 – 1 – 2 – 3 – 4 – 5 – 6  not at all very much |
|  | 42* | *Today I was able to face my problems.* | 0 – 1 – 2 – 3 – 4 – 5 – 6  not at all very much |
|  |  |  |  |
|  |  | Personal comment | Open text field |

Welcome and thank you for your participation in the therapeutic feedback.

Please fill in the following questionnaire according to the experiences you had today.

Some of the items concern the progress of your therapy or the relationship to therapists and other patients. Even if you did not have direct contact with therapists today, please answer the questions according to how your experiences, impressions and mood made you feel today.

Thank you!
